# Fgf21 regulates T-cell development in the neonatal and juvenile thymus

**DOI:** 10.1038/s41598-017-00349-8

**Published:** 2017-03-23

**Authors:** Yoshiaki Nakayama, Yuki Masuda, Hiroya Ohta, Tomohiro Tanaka, Miwa Washida, Yo-ichi Nabeshima, Ayumi Miyake, Nobuyuki Itoh, Morichika Konishi

**Affiliations:** 1 Department of Microbial Chemistry, Kobe Pharmaceutical University, Kobe, Japan; 2 Department of Molecular Medicine and Metabolism, Research Institute of Environmental Medicine, Nagoya University, Nagoya, Japan; 3 Medical Innovation Center, Graduate School of Medicine, Kyoto University, Kyoto, Japan; 4 Laboratory of Molecular Life Science, Foundation for Biomedical Research and Innovation, Kobe, Hyogo Japan; 5 Department of Genetic Biochemistry, Kyoto University Graduate School of Pharmaceutical Sciences, Sakyo, Kyoto Japan

## Abstract

We have previously shown that *Fibroblast growth factor 21* (*Fgf21*) is expressed in the thymus as well as in the liver. In line with this expression profile, Fgf21 was recently reported to protect against ageing-related thymic senescence by improving the function of thymic epithelial cells (TECs). However, the function of Fgf21 in the juvenile thymus remained to be elucidated. We investigated the physiological roles of Fgf21 in the juvenile thymus and found that young *Fgf21* knockout mice, but not *β-Klotho* knockout mice nor adult *Fgf21* knockout mice, showed a significant reduction in the percentage of single-positive CD4^+^ and CD8^+^ thymocytes without obvious alteration in TECs. Furthermore, treatment with recombinant FGF21 protein rescued the impairment in fetal thymus organ culture (FTOC) of *Fgf21* knockout mice. Annexin V staining revealed FGF21 protein enhanced apoptosis of immature thymocytes undergoing selection process in FTOC, suggesting that FGF21 may facilitate the selection of developing T cells. Endocrine Fgf21 from the liver induced by metabolic stimulation did not affect juvenile thymocyte development. Our data suggest that Fgf21 acts as one of intrathymic cytokines in the neonatal and juvenile thymus, involving thymocyte development in a β-Klotho-independent manner.

## Introduction

The fibroblast growth factor (Fgf) family consists of 22 human polypeptide growth factors that regulate various biological processes, including mitogenesis, cellular differentiation, and metabolism^1^. Recently, Fgf21 has been attracting attention as a drug candidate for the treatment of obesity and metabolic syndrome^2^. Pharmaceutical administration of recombinant FGF21 protein or FGF21 mimetics is expected to improve obesity, obesity-associated hyperglycaemia, and hyperlipidaemia^3, 4^. Physiologically, Fgf21 is produced mainly by liver, adipose, and muscle tissues^5–7^. Secreted Fgf21 could regulate whole-body metabolism via its impact on the function of several tissues including brain and adipose tissues in an endocrine manner.

Previously we have shown that *Fgf21* is expressed in the mouse thymus and liver^8^. The thymus is one of the primary lymphoid tissues, where progenitor cells originating from the bone marrow develop into mature T lymphocytes. The developmental process of the T lymphocytes in the thymus is highly regulated, and the expression of different cell surface markers is used to identify the different stages of T lymphocytes. For example, progenitor cells migrated from bone marrow lack the expression of CD4 and CD8 (termed as double negative cells: DN cells). These DN cells develop into both CD4 and CD8 positive cells (DP cells), followed by mature CD4 positive and CD8 negative cells (termed as CD4 single positive cells: CD4SP cells) or CD8 positive and CD4 negative cells (termed as CD8 single positive cells: CD8SP cells).

This developmental process of T lymphocytes is supported by two types of thymic epithelial cells (TECs)^9, 10^. Cortical TECs (cTECs) in the cortex of the thymus are necessary for the positive selection of immature DP cells^11, 12^. Immature DP cells are positively selected by recognition of self-peptide-MHC complexes on cTECs, while the remaining DP cells undergo apoptosis by neglect. Positively selected DP cells then differentiate into CD4SP or CD8SP cells and migrate to the medulla, where medullary TECs (mTECs) expressing the transcription factor Aire (Autoimmune regulator) are expanded. Aire enables the expression of tissue-specific antigens in mTECs. CD4SP or CD8SP cells, which bind strongly to the endogenous tissue-specific antigens presented on mTECs, are negatively selected and die via apoptosis.

The expression of *Fgf21* in the thymus suggests that Fgf21 might play potential roles in thymic physiology. In line with this prediction, Youm *et al*. have recently shown that Fgf21 protects thymic senescence by improving the function of TECs^13^. In this report, Youm *et al*. focused on the effects of Fgf21 against the thymic involution caused by ageing. Therefore, they examined the aged thymus of *Fgf21* transgenic and knockout mice. However, the function of Fgf21 in the juvenile thymus remained to be elucidated. Preliminary findings showed that Fgf21 is expressed in the mouse thymus from the fetal to juvenile stages, suggesting that Fgf21 is involved in thymic development or function in juveniles in addition to inhibition of thymic involution. In this study, we investigated the physiological roles of Fgf21 in thymic physiology in juveniles using *Fgf21* knockout mice and fetal thymus organ culture (FTOC).

## Results

### Fgf21 is enriched in mature mTECs

**Figure 1:**
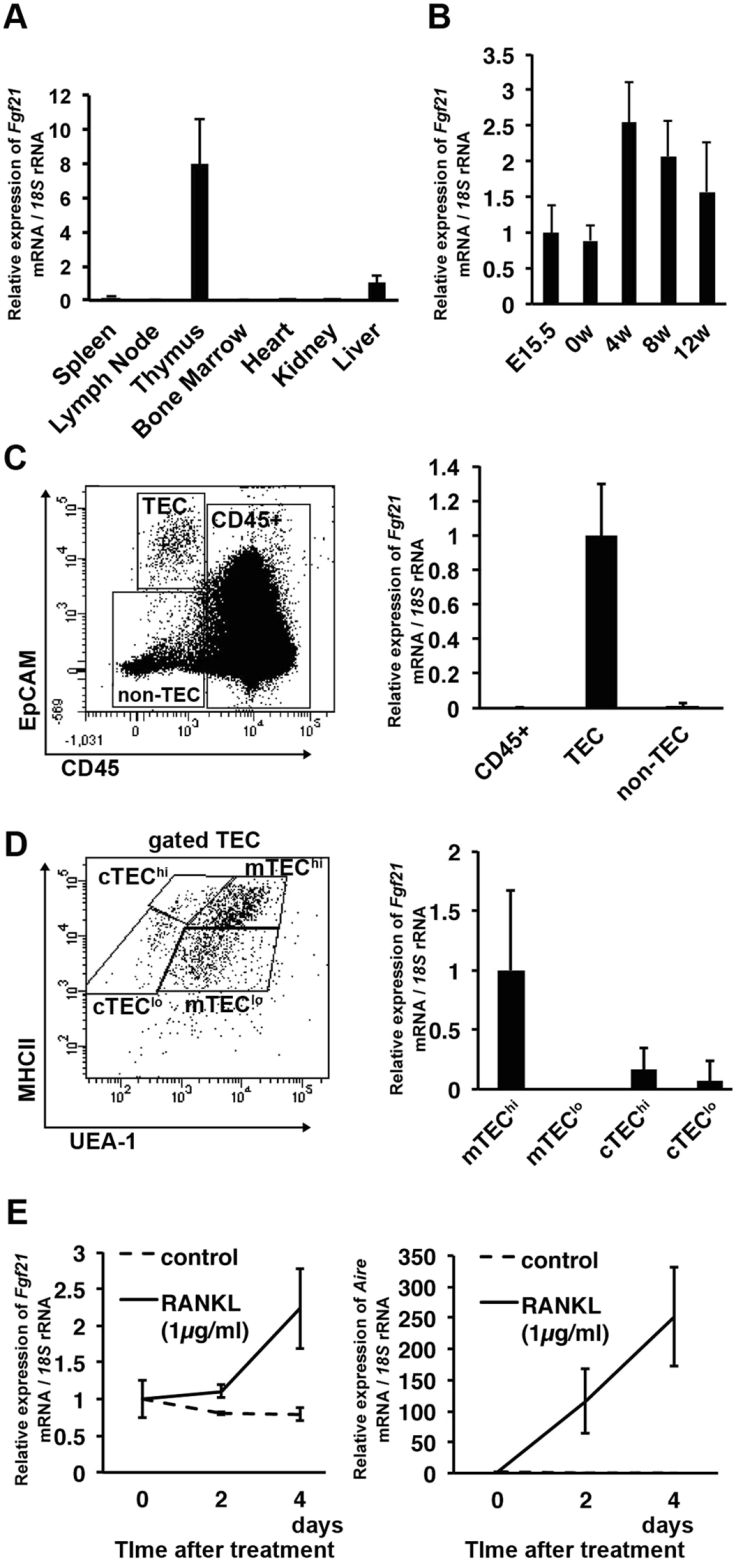
*Fgf21* mRNA is enriched in mature mTECs. (**A** and **B**) Expression levels of *Fgf21* mRNA in the various tissues from 4-week-old C57BL/6 mice (**A**) and in the thymus of E15.5 and 0-, 4-, 8- and 12-week-old (**B**). Relative *Fgf21* mRNA expression was normalised against *18S* rRNA expression. (**C** and **D**) Thymic cell suspensions from 4-week-old C57BL/6 mice were assessed by flow cytometric analysis using anti-CD45 mAb, anti-EpCAM mAb, anti-MHC II (I-A/I-E) mAb, and UEA-1 lectin, and expression levels of *Fgf21* mRNA were determined by RT-realtime PCR in the subpopulations of thymic cells from 4-week-old C57BL/6 mice. (**C**) Anti-CD45 and anti-EpCAM staining discriminated CD45^+^, TEC (CD45^−^EpCAM^+^), and non-TEC stromal cell (CD45^−^EpCAM^−^) subpopulations. (**D**) Gated TECs were subdivided into cTEC^hi^ (UEA-1^−^MHC^high^), cTEC^lo^ (UEA-1^−^MHC^low^), mTEC^hi^ (UEA-1^+^MHC^high^), and mTEC^lo^ (UEA-1^+^MHC^low^) subpopulations. (**E**) Thymocytes were removed from embryonic thymi by 1.35 mM dGuo treatment for 6 days, and remaining stromal cells were stimulated with 1 μg/ml RANKL to induce the maturation of TECs. *Fgf21* and *Aire* mRNA were increased along with TEC maturation. Graphs represent the mean ± SD; n ≧5 replicates per group from at least 2 independent experiments.

We previously showed that *Fgf21* mRNA was abundantly expressed in the liver and thymus of adult mice^8^. In 4-week-old male mice or mouse embryos at day 15.5 of gestation (E15.5), *Fgf21* expression was detected in the thymus (Fig. 1A and B). The thymus mainly consists of a large population of thymocytes and a small number of TECs, fibroblasts, and blood vessels, where thymocytes differentiate into mature T lymphocytes through the interaction with TECs. To investigate which cell populations in the thymus express Fgf21, the expression level of *Fgf21* mRNA was analysed by quantitative RT-PCR combined with cell sorting. Single-cell suspensions of enzymatically digested thymus were stained with anti-CD45 and anti-EpCAM monoclonal antibody (mAb) and sorted by flow cytometry into three fractions (CD45^+^ thymocytes; CD45^+^, TEC; CD45^−^EpCAM^+^, and non-TEC stromal cells; CD45^−^EpCAM^−^) (Fig. 1C). Quantitative RT-PCR using these fractions revealed that *Fgf21* mRNA was exclusively expressed in the TEC fraction (Fig. 1C). We further analysed the expression levels of *Fgf21* mRNA in the TEC subsets defined by UEA-1 staining and MHCII levels; cTEC^hi^ (UEA-1^−^MHC^high^), cTEC^lo^ (UEA-1^−^MHC^low^), mTEC^hi^ (UEA-1^+^MHC^high^), and mTEC^lo^ (UEA-1^+^MHC^low^) subpopulations (Fig. 1D)^14^. *Fgf21* mRNA was abundantly expressed in mTEC^hi^, and at low levels in cTEC^hi^ and cTEC^lo^ subsets (Fig. 1D), suggesting that *Fgf21* is predominantly expressed in mature mTECs. Therefore, we next examined the relationship between expression levels of *Fgf21* mRNA and the maturation of TECs using FTOC. Thymocytes were removed from E15.5 thymi by 1.35 mM dGuo treatment for 6 days, and the remaining stromal cells were stimulated with 1 μg/ml RANKL for 4 days to induce the maturation of TECs^15^. *Fgf21* and *Aire* mRNA expression was determined every 2 days after RANKL treatment. Along with the maturation of TECs indicated by the induction of *Aire* mRNA, *Fgf21* mRNA was also increased (Fig. 1E).

### *Fgf21* knockout mice show decreased thymocyte development in the neonatal stage

**Figure 2:**
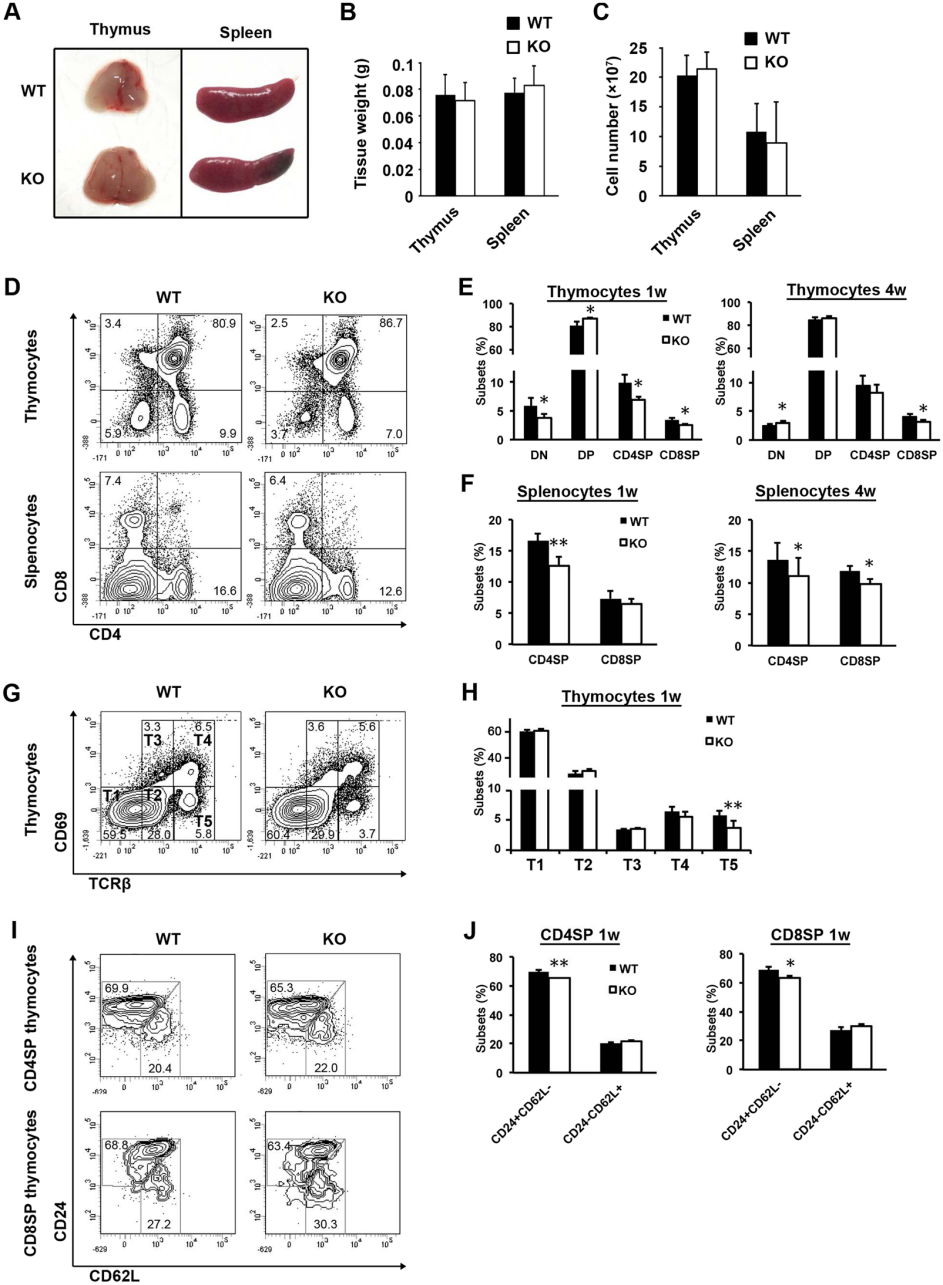
CD4SP and CD8SP populations are decreased in *Fgf21* KO mice. (**A** and **B**) Thymi and spleens were isolated from 4-week-old WT and *Fgf21* KO mice, and their weights were measured. (**C**) Cell numbers of enzymatically digested suspensions from thymi and spleens were counted. (**D**) Thymocytes and splenocytes from WT and *Fgf21* KO mice were stained with anti-CD4 and anti-CD8 mAb. Representative flow cytometry plots show CD4/CD8 analysis of thymocytes and splenocytes from 1-week-old WT and *Fgf21* KO mice. (**E**) Charts show the percentage of DN, DP, CD8SP, and CD4SP cells from 1- and 4-week-old WT and *Fgf21* KO mice. (**F**) Charts show the percent of CD4SP and CD8SP cells from 1- and 4-week-old WT and *Fgf21* KO mice. (**G**) Thymocytes from 1-week-old WT and *Fgf21* KO mice were defined by TCRβ and CD69 levels and subdivided into 5 subsets (T1; TCRβ^−^CD69^−^, T2; TCRβ^int^CD69^−^, T3; TCRβ^int^CD69^+^, T4; TCRβ^hi^CD69^+^, and T5; TCRβ^hi^CD69^−^). (**H**) Charts show the percentage of the 5 subsets from 1-week-old WT and *Fgf21* KO mice. (**I**) Gated CD4SP and CD8SP cells from 1-week-old WT and *Fgf21* KO mice were defined by CD62L and CD24 levels and subdivided into CD24^+^CD62L^−^ immature and CD24^−^CD62L^+^ mature SP cells. (**J**) Charts show the percentage of CD24^+^CD62L^−^ immature and CD24^−^CD62L^+^ mature SP subsets from 1-week-old WT and *Fgf21* KO mice. All data shown are the mean ± SD from ≧6 mice per genotype from 2 independent experiments. *P < 0.05, *P < 0.01 versus WT mice.

The enriched expression of *Fgf21* in mature TECs led us to examine whether Fgf21 plays an intrinsic role in thymocyte development. We compared the thymi and spleens of wild-type (WT) and *Fgf21* knockout (KO) mice (Fig. 2A). Weights and cellularity of these tissues from 4-week-old male mice were similar to one another (Fig. 2B and C). CD4/CD8 double staining of the thymocytes revealed that the percentage and cellularity of CD4SP and CD8SP cells at 1- and 4-weeks-old was significantly or tended to be decreased in *Fgf21* KO mice compared to that of WT mice (Fig. 2D,E, and S1A). Additionally, the percentage and cellularity of CD4SP and CD8SP splenocytes at 1- and 4-week-old correspondingly decreased in the secondary lymphoid organ of *Fgf21* KO mice (Fig. 2D,F, and S1B). We further assessed the thymocytes of *Fgf21* KO mice using anti-TCRβ and anti-CD69 mAb to determine which developmental stages of thymocytes Fgf21 affects (Fig. 2G, T1; TCRβ^−^CD69^−^, T2; TCRβ^int^CD69^−^, T3; TCRβ^int^CD69^+^, T4; TCRβ^hi^CD69^+^, and T5; TCRβ^hi^CD69^−^)^16^. The later developmental stages of thymocytes (T4 and T5) decreased, whereas that of immature thymocytes (T1, T2, and T3) increased (Fig. 2G,H and S1C). Expression analysis of CD24 and CD62L revealed that *Fgf21* KO mice exhibited the reduction in the percentage, but not in the absolute number, of CD24^+^CD62L^−^ immature CD4SP and CD8SP cells (Fig. 2I,J and S1D). These data suggest that Fgf21 might regulate the later stage of thymocyte development at the transition from double-positive to single-positive thymocyte in the neonatal.

### Fgf21 is not necessary for the proliferation and organization of TECs

**Figure 3:**
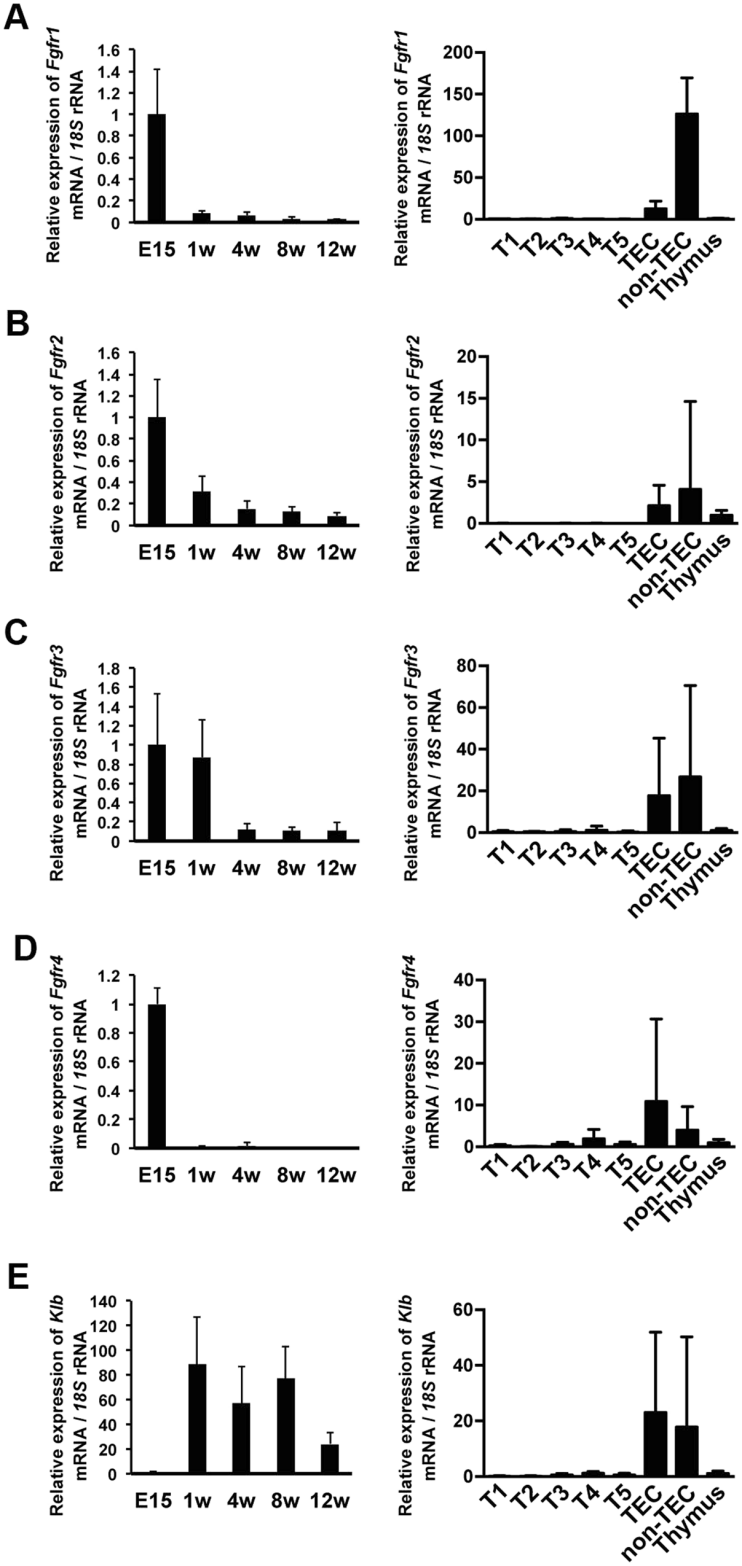
*Fgfr* and *Klb* are highly expressed in thymic stromal cells. (**A–E**) Relative mRNA expression of *Fgfr1* (**A**), *Fgfr2* (**B**), *Fgfr3* (**C**), *Fgfr4* (**D**), and *Klb* (**E**) in the thymus of E15.5 and 1-, 4-, 8- and 12-week-old (left panels), and in thymocyte subpopulations (T1-T5), TECs (CD45^−^EpCAM^+^), and non-TEC stromal cells (CD45^−^EpCAM^−^) of 4-week-old C57BL/6 mice (right panels). Graphs represent the mean ± SD; n ≧ 5 replicates per group from at least 3 independent experiments.

Fgf21 signalling is thought to require Fgfr and its co-receptor, β-Klotho (Klb)^17–19^. We therefore examined the mRNA expression of these receptors in the developmental stages of thymus and different cell types. *Fgfr1-4* were abundantly expressed in the thymus at E15.5 and decreased along with thymic growth, whereas *Klb* were only expressed after birth (Fig. 3A–E). All of these receptors highly expressed in TECs and other cell types, but little to no expression was detected in thymocytes at all developmental stages (Fig. 3A–E). These results indicate that Fgf21 might interact with TECs and other thymic stromal cells.

**Figure 4:**
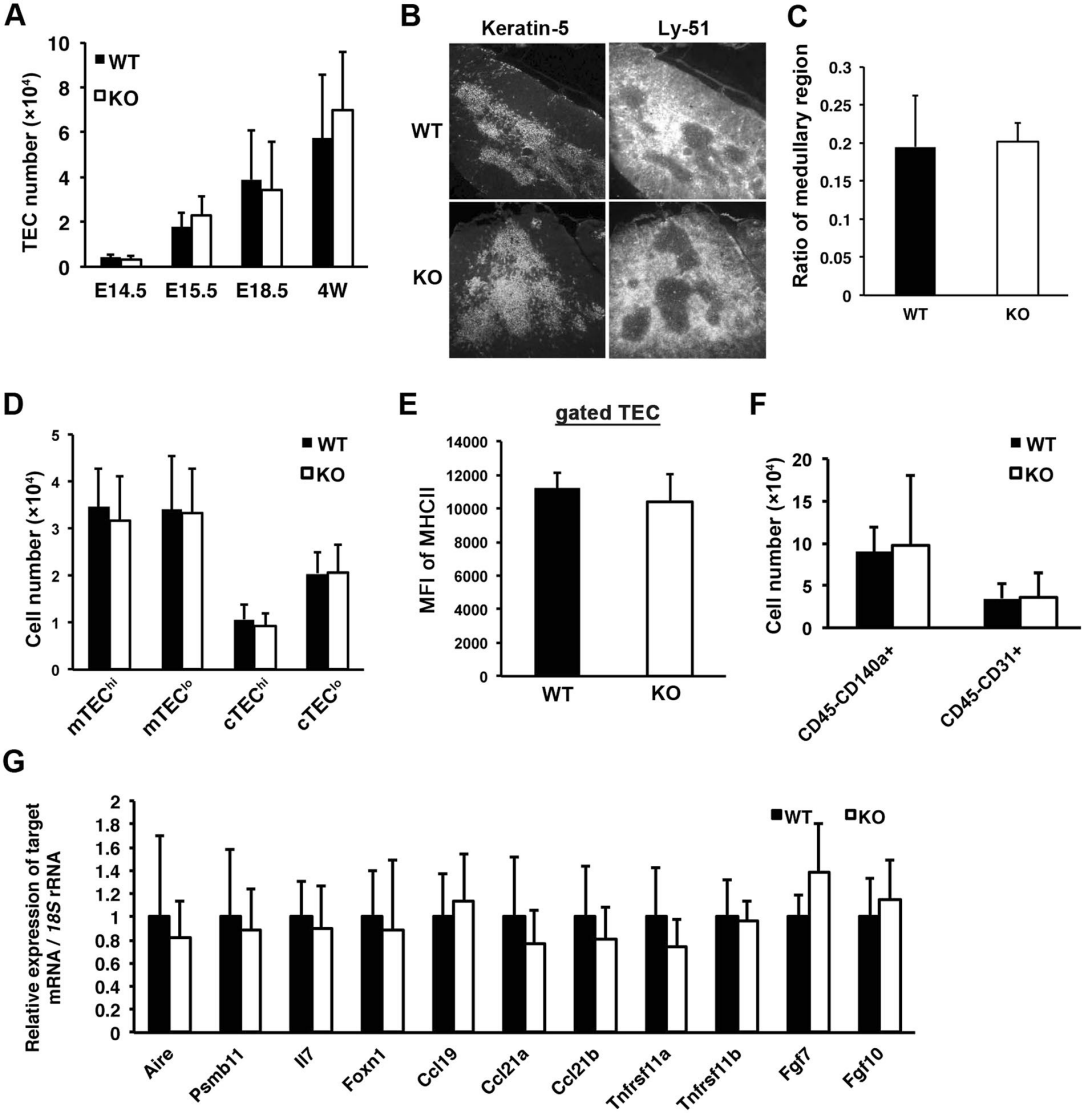
Thymic stromal cells are not obviously altered in *Fgf21* KO mice. (**A**) Enzymatically digested thymic cell suspensions were stained with anti-CD45 mAb and anti-EpCAM mAb, and the TEC (CD45^−^EpCAM^+^) number was counted from the thymi of E14.5, E15.5, E18.5, and 4-week-old WT and *Fgf21* KO mice. (**B**) Immunohistochemistry of WT and *Fgf21* KO thymi with anti-Keratin-5 antibody for a medullary TEC marker (left panels) and anti-Ly-51 antibody for a cortical TEC marker (right panels). (**C**) The ratio of Keratin-5-positive thymic medullary regions was measured from immunostained sections. (**D**) Gated TECs of 1-weeks old were subdivided into mTEC^hi^ (Ly51^−^MHC^high^), mTEC^lo^ (Ly51^−^MHC^low^), cTEC^hi^ (Ly51^+^MHC^high^), and cTEC^lo^ (Ly^+^MHC^low^) subpopulations. Graphs represent the percentage of subpopulations. (**E**) The mean fluorescence intensity (MFI) of surface MHCII on the TEC was measured by flow cytometry with anti-MHCII antibody. (**F**) Gated CD45^−^ thymic stromal cells of 1-weeks old were stained with anti-CD140a and anti-CD31 antibodies. Graphs represent the cellularity of CD45^−^CD140a^+^ fibroblasts and CD45^−^CD31a^+^ endothelial cells in the thymus of WT and *Fgf21* KO mice. (**G**) The expression levels of thymic factors were measured in the thymus of 1-week-old WT and *Fgf21* KO mice. Graphs represent the mean ± SD; n ≧ 5 replicates per group from at least 3 independent experiments.

As shown in our results, Fgf21 receptors were expressed predominantly in thymic stromal cells. In addition, Fgf21 was reported to be important for the absolute number of TECs as well as of thymocytes in aging mice^13^. Therefore, we investigated whether Fgf21 affects the composition of TECs in the neonatal and juvenile thymus. Cellularity of CD45^−^EpCAM^+^ TEC was determined along the development of the thymus. There was no obvious difference between the cellularity of WT and KO mice (Fig. 4A). We next examined the organization of medullary and cortex TECs in *Fgf21* KO mice. Immunostaining of thymic sections with anti-Keratin-5 for mTECs and anti-Ly-51 for cTECs revealed that the ratio of mTEC in *Fgf21* KO was similar to that of the WT (Fig. 4B and C). The distribution of mTECs in the thymus was also obviously unchanged in *Fgf21* KO mice (Fig. 4C). Flow cytometry analysis exhibited similar distribution of TEC subpopulations (Fig. 4D and S2A), CD45^−^CD140a^+^ fibroblasts, and CD45^−^CD31^+^ endothelial cells (Fig. 4F and S2B), and comparable MHCII levels on the TEC (Fig. 4E). Moreover, expression levels of *Aire* and *Psmb11* (*β5t*) mRNA, which are markers for medullary and cortex TECs, respectively, were not different between the thymi of WT and *Fgf21* KO mice (Fig. 4G). The expression levels of genes involved in thymic functions, such as *Il7, Ccl19, Ccl21a, Ccl21b*, and *Foxn1*, were similar to each other (Fig. 4G). Taken together, these results imply that Fgf21 may not affect the proliferation and organization of TECs.

### β-Klotho is not involved in thymocyte development

**Figure 5:**
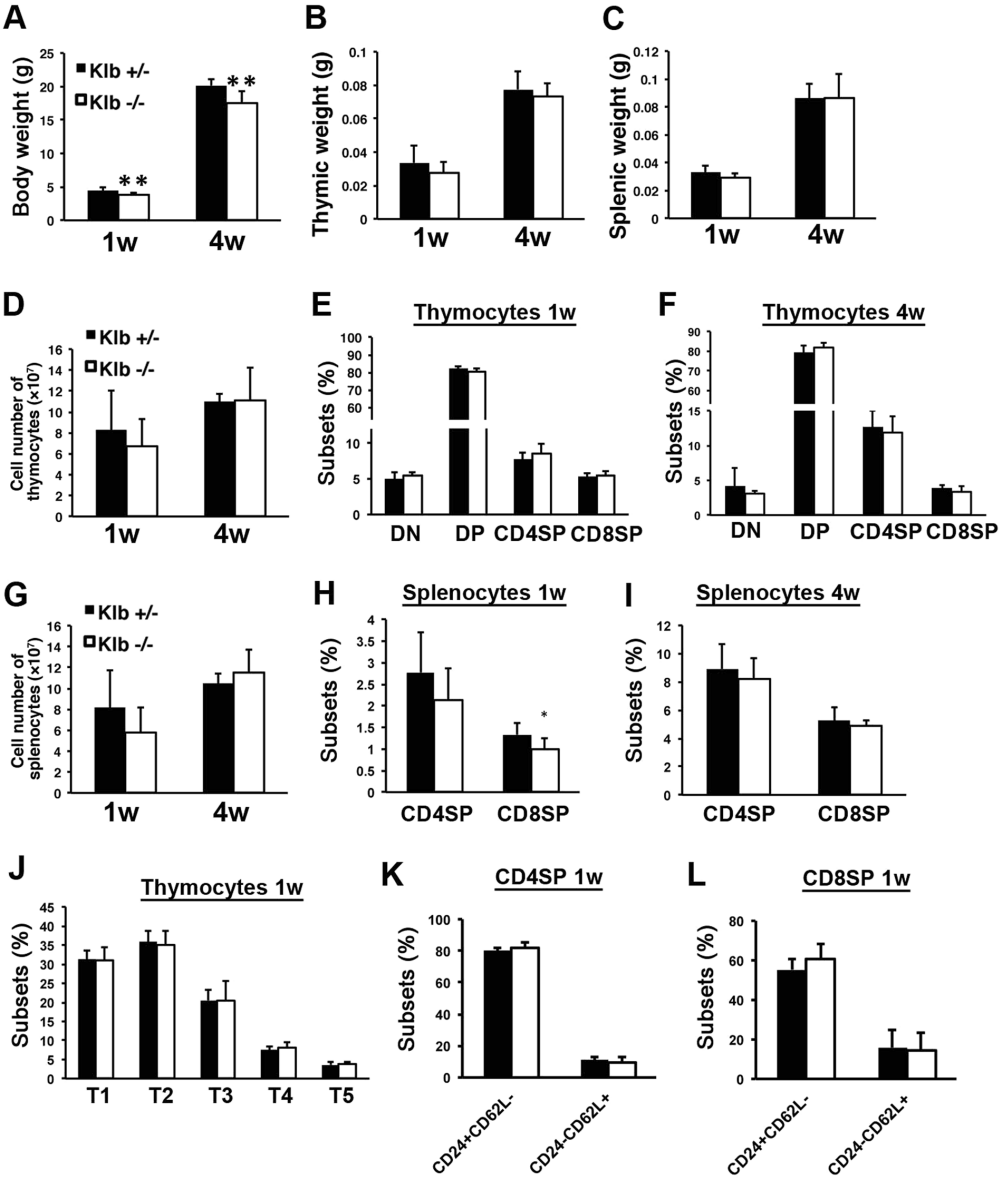
*Klb* is not involved in the development of thymocytes. (**A–C**) Body, thymic, and splenic weights of 1- and 4-week-old *Klb* ^+/−^ and *Klb* ^−/−^ mice. *Klb* ^−/−^ mice showed decreased body weight compared to *Klb* ^+/−^ mice. (**D** and **G**) Cell numbers of enzymatically digested suspensions from thymi and spleens were counted. (**E,F,H** and **I**) Thymocytes and splenocytes from 1- and 4-week-old *Klb* ^+/−^ and *Klb* ^−/−^ mice were stained with anti-CD4 and anti-CD8 mAb. Charts show the percent of DN, DP, CD8SP, and CD4SP from 1- and 4-week-old *Klb* ^+/−^ and *Klb* ^−/−^ mice. (**J**) Thymocytes from 1-week-old *Klb* ^+/−^ and *Klb* ^−/−^ mice were defined by TCRβ and CD69 levels and subdivided into 5 subsets. Charts show the percentage of the 5 subsets from 1-week-old *Klb* ^+/−^ and *Klb* ^−/−^ mice. (**K** and **L**) Charts show the percentage of CD24^+^CD62L^−^ immature and CD24^−^CD62L^+^ mature SP subsets from gated CD4SP (**K**) and CD8SP cells (**L**) of 1-week-old *Klb* ^+/−^ and *Klb* ^−/−^ mice. All data shown are the mean ± SD from ≧8 mice per genotype. *P < 0.05, *P < 0.01 versus *Klb* ^+/−^ mice.

β-Klotho is known to be a co-receptor for mediating the signals of endocrine FGFs^17^. To investigate whether β-Klotho was involved in the functions of Fgf21 in the thymus, we examined the population of thymocytes and splenocytes from *Klb* ^−/−^ mice. As shown previously^20^, the body weight of *Klb* ^−/−^ mice was smaller than that of *Klb* ^+/−^ mice (Fig. 5A), and tissue weights and cellularity of the thymus and spleen, accordingly, had decreased tendencies especially in 1-week-old *Klb* ^−/−^ mice (Fig. 5B–D,G, and S3). Nevertheless, the ratios of CD4SP and CD8SP cells in the thymus from 1- and 4-week-old mice were not different between *Klb* ^+/−^ and *Klb* ^−/−^ mice (Fig. 5E and F). On the other hand, in the spleen, CD8SP cells at 1-week of age were significantly decreased, and CD4SP cells tended to decrease in *Klb* ^−/−^ mice compared to those of *Klb* ^+/−^ mice (Fig. 5H). These differences were not observed in 4-week-old *Klb* ^+/−^ and *Klb* ^−/−^ mice (Fig. 5I). The percentage of thymocyte developmental subpopulations defined by TCRβ/CD69 and CD24/CD62L staining were not different between *Klb* ^+/−^ and *Klb* ^−/−^ mice (Fig. 5J–L and S3E–G). These data suggest that β-Klotho is not involved in the development of thymocytes.

### Fgf21 regulates thymocyte differentiation in FTOC

**Figure 6:**
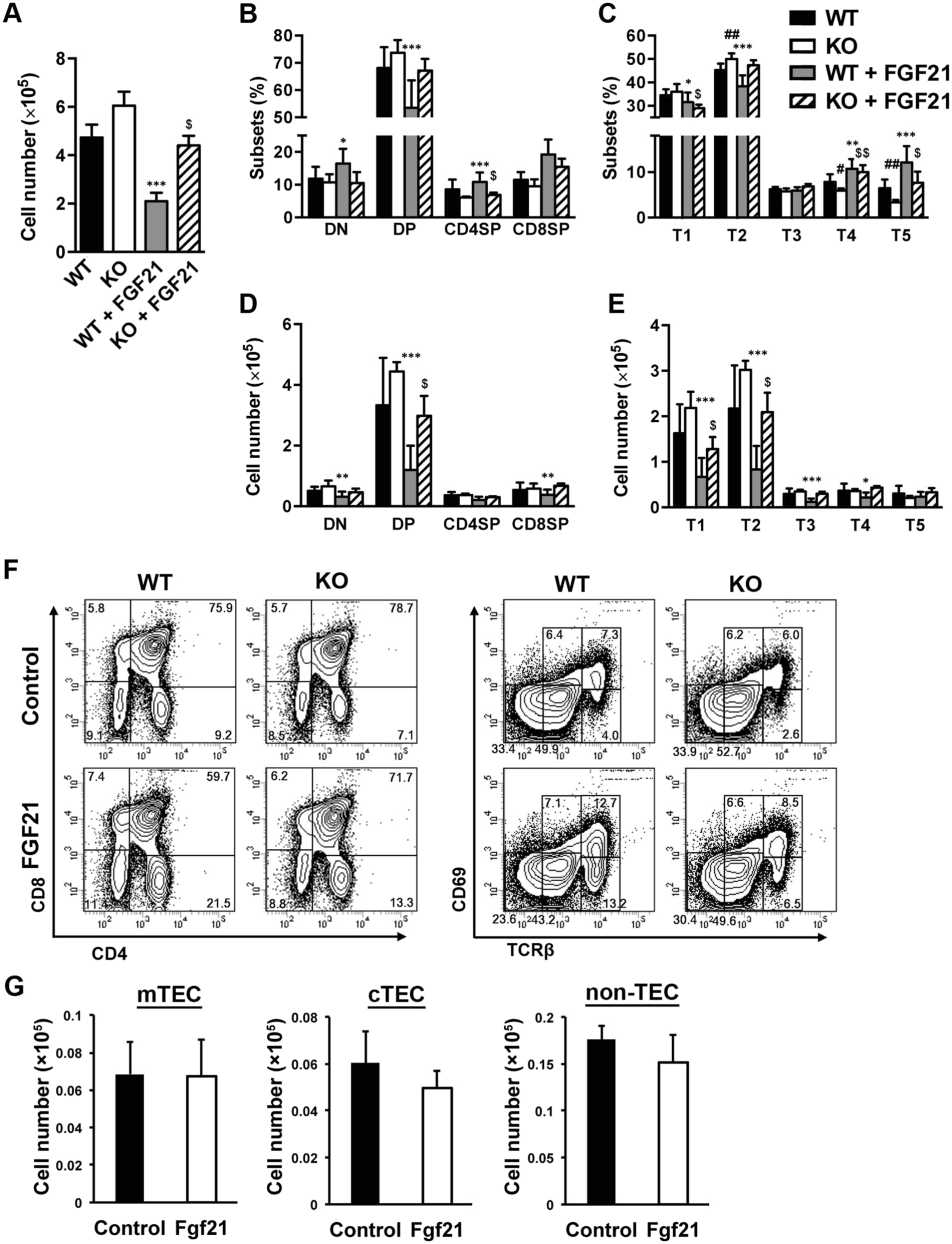
FGF21 treatment alters T-cell development in FTOC. WT and *Fgf21* KO foetal thymi were cultured with or without recombinant human FGF21 protein (500 ng/ml) for 14 days. (**A**) Total cell numbers of thymocytes in FTOCs are presented. Flow cytometry analysis of CD4/8 (**B**) and TCRβ/CD69 (**C**) distribution and thymocyte subset number (**D** and **E**). (**F**) Representative flow cytometry data for each FTOC. (**G**) Numbers of mTEC (CD45^−^EpCAM^+^UEA-1^+^), cTEC (CD45^−^EpCAM^+^UEA-1^−^), and non-TEC stromal cells (CD45^−^EpCAM^−^) are shown. Data represents the mean ± SD of two separate experiments. ^*,#^P < 0.05, ^**,##^P < 0.01, and ^***^P < 0.001 versus WT without rhFGF21, ^$^P < 0.05, and ^$$^P < 0.01 versus *Fgf21* KO without rhFGF21.

To confirm the role of FGF21 during thymocyte development, we examined the effects of FGF21 on embryonic thymus growth in FTOCs. E15.5 thymic lobes were cultured in the presence or absence of recombinant human FGF21 (rhFGF21) protein, and thymocyte numbers were assessed after 14-day of culture by flow cytometry. We observed that rhFGF21 treatment significantly decreased total thymocyte cellularity in WT FTOCs (Fig. 6A). Both the percentage and the absolute number of WT DP thymocytes were also drastically decreased by rhFGF21 treatment (Fig. 6B,D and F). When thymocytes were subdivided on the basis of TCR and CD69 expression, the percentages of the most immature pre-selected thymocytes (T1 and T2) were significantly decreased in WT FTOCs supplemented with rhFGF21, while the percentages of postpositive-selected thymocytes (T4) and mature thymocytes (T5) were significantly increased (Fig. 6C and F). Analysis of the absolute cell number of each developmental stage shows that WT FTOCs supplemented with rhFGF21 significantly decreased immature thymocyte (T1, T2, T3, and T4) numbers, but did not greatly affect mature thymocyte (T5) numbers (Fig. 6E).

Consistent with observations in 1- and 4-week-old mice, we observed a significant reduction in the percentages of T4 and T5 cells at later stages of T cell development in *Fgf21* KO FTOCs (Fig. 6C and F). Supplementation with rhFGF21 recovered the reduction of these cells in *Fgf21* KO FTOCs to a similar extent of those in WT FTOCs. These results suggest that Fgf21 regulates thymocyte development. As rhFGF21 treatment decreases the number of immature thymocytes but does not affect the number of mature thymocytes, FGF21 may influence apoptosis of immature thymocytes.

Since our results showed that *Fgfr1-4* and *Klb* are highly expressed in TECs and other thymic stromal cells, but that *Fgf21* KO mice did not show obvious changes in TECs (Figs 3 and 4), we examined the direct effect of FGF21 on the proliferation of TECs. For this, thymic lobes were first cultured in the presence of 2-deoxyganosine (2DG-FTOC). After treatment with 2DG-FTOC for 4 days, the thymic lobes were re-cultured with rhFGF21 for 4 additional days. Supplementing 2DG-FTOC with rhFGF21 did not affect the number of mTECs, cTECs or non-TEC stromal cells (Fig. 6G and S2C). These results, taken together with the results from 4-week-old *Fgf21* KO mice, suggest that Fgf21 has no direct effect on the proliferation or differentiation of TECs.

### FGF21 increases apoptosis of immature thymocytes undergoing selection processes

**Figure 7:**
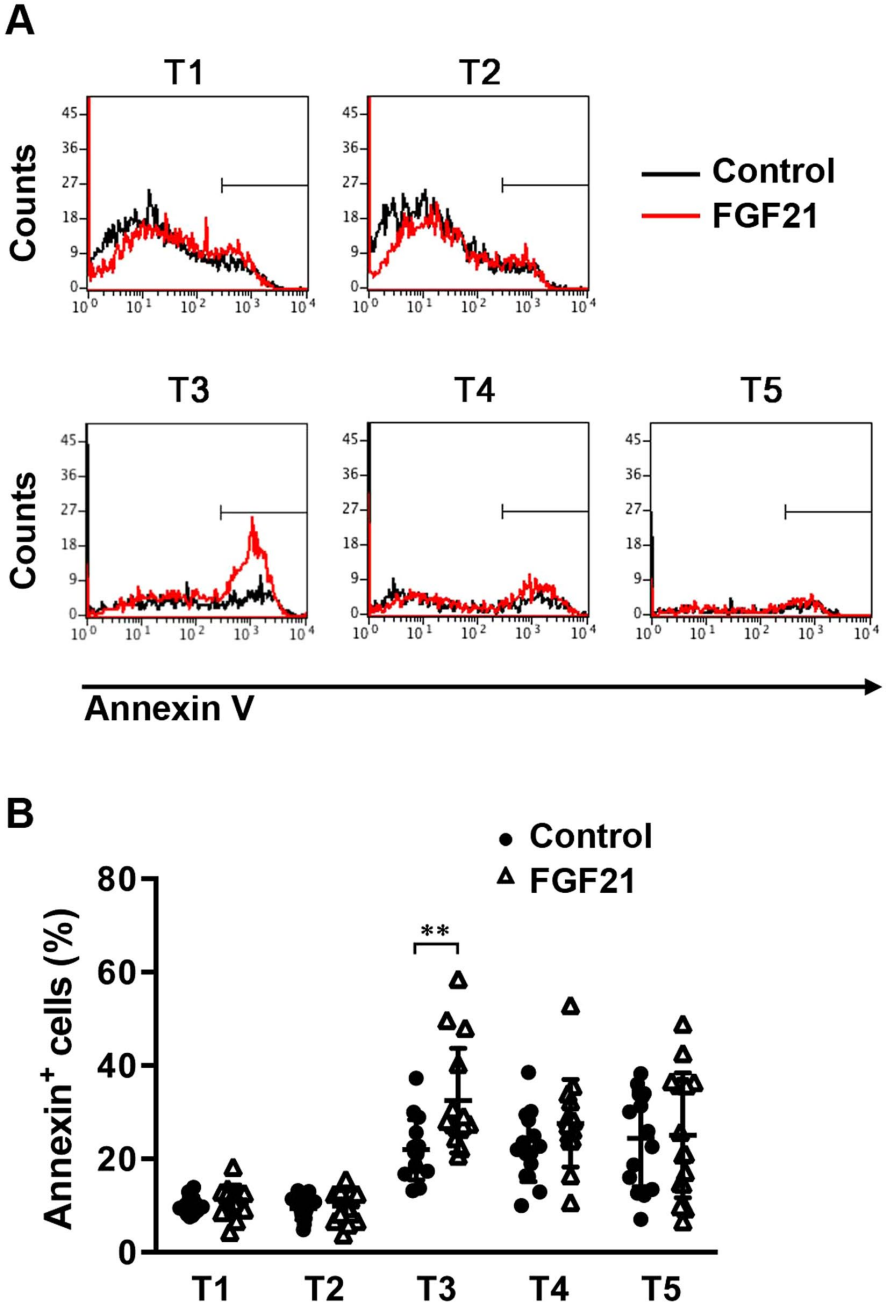
FGF21 treatment results in increased apoptosis of immature thymocytes. FTOC was performed in the presence or absence of recombinant human FGF21 (500 ng/ml) for 14 days. Annexin V staining was performed to detect apoptotic T cells by flow cytometry. (**A**) Representative histograms show the percentage of Annexin V^+^ cells in gated thymocyte subsets. (**B**) Graph showing the analysis of the percentage of Annexin V^+^ cells in thymocytes from FTOCs. All data shown are the mean ± SD. **P < 0.01 versus control.

Signalling that is mediated by interaction of TCR with self-peptide-MHC ligand complexes leads to the fate decision of DP thymocytes^21–23^. Too little signalling (death by neglect) or too much signalling (negative selection) results in apoptosis. The intermediate level of TCR signalling allows successful differentiation (positive selection). As rhFGF21 treatment decreased the number of immature thymocytes in FTOCs, we examined whether FGF21 treatment induces thymocyte apoptosis in FTOCs using annexin V staining. At day 14 of culture, rhFGF21 treatment specifically increased the percentages of early apoptotic, annexin V^+^ cells in T3 thymocytes undergoing selection (Fig. 7A and B). This result suggests that enhanced apoptosis of thymocytes in rhFGF21-treated thymic lobes might be the mechanism responsible for the reduced number of thymocytes observed during FTOCs. This result also suggests that FGF21 treatment may facilitate the selection of developing T cells in FTOCs without providing early committed T cells from bone marrow, resulting in a decrease in the number of immature thymocytes.

### Fgf21 induced by protein-free diet does not involved in thymocyte alteration

**Figure 8:**
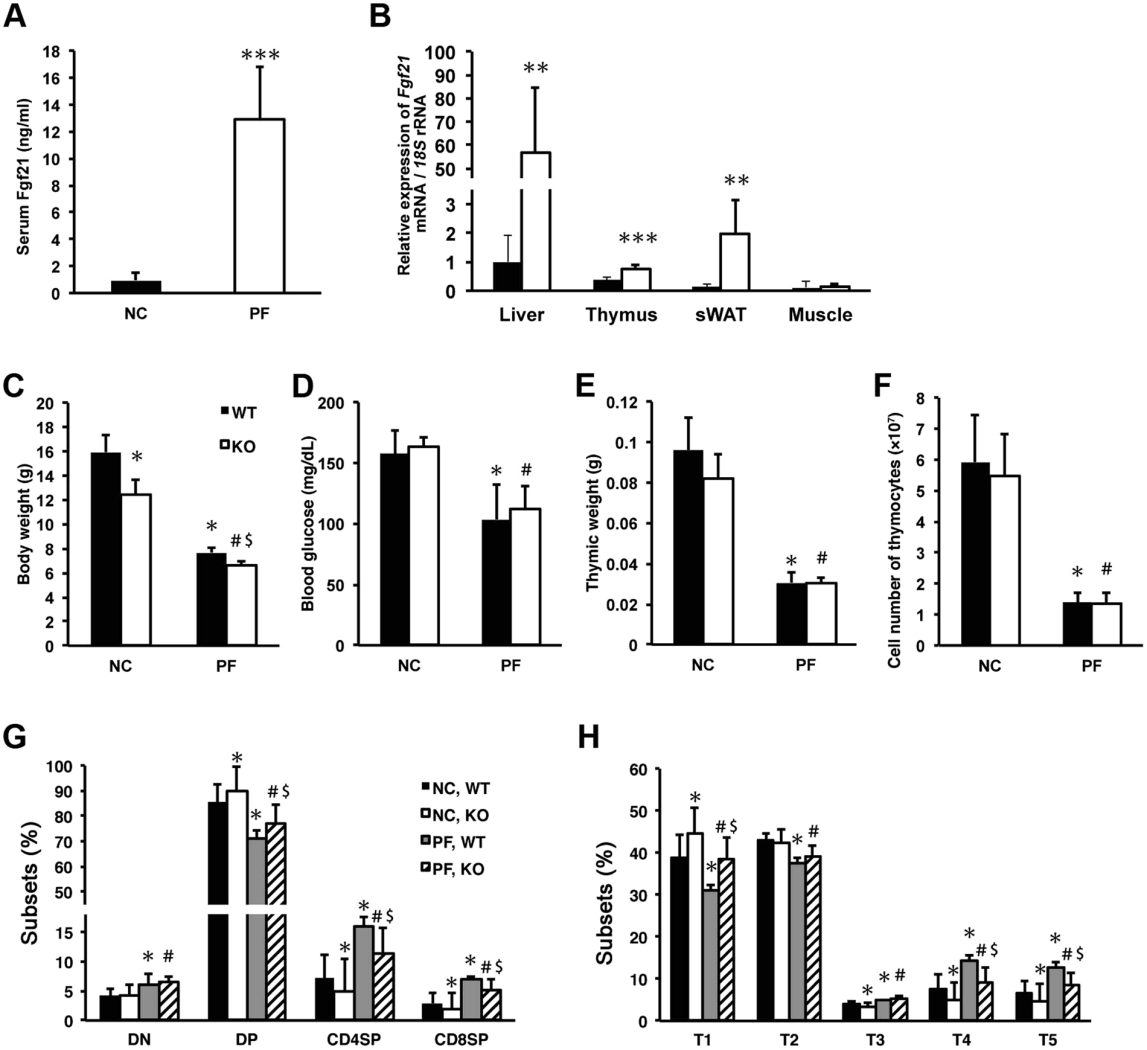
Fgf21 induced by protein-free diet does not affect thymic change by protein malnutrition. (**A**) Serum Fgf21 levels of 4-week-old C57BL/6 mice fed for 1 week with normal chow diet (NC) or protein-free diet (PF) were determined with ELISA assays. Data shown are the mean ± SD from ≧6 mice. ^**^P < 0.001 versus NC. (**B**) Relative expression levels of *Fgf21* mRNA in the liver, thymus, subcutaneous white adipose tissue (sWAT), and skeletal muscle from mice fed with NC or PF. Data shown are the mean ± SD from ≧6 mice. ^**^P < 0.01, ^**^P < 0.001 versus NC. (**C–F**) Body weight (**C**), blood glucose (**D**), thymic weight (**E**), and thymic cell number (**F**) of WT and *Fgf21* KO mice fed with NC or PF. Data shown are the mean ± SD from ≧5 mice. ^*^P < 0.05, ^#^P < 0.05 versus WT and *Fgf21* KO mice fed with NC, respectively. ^$^P < 0.05 versus WT mice fed with PF. (**G** and **H**) Thymocytes from WT and *Fgf21* KO mice fed with NC or PF were stained with anti-CD4 and anti-CD8 mAb (**G**), or anti-TCRβ and anti-CD69 mAb (**H**). All data shown are the mean ± SD from ≧5 mice. ^*^P < 0.05, ^#^P < 0.05 versus WT and *Fgf21* KO mice fed with NC, respectively. ^$^P < 0.05 versus WT mice fed with PF.

Fgf21 is known as a metabolic regulator that is produced mainly in the liver and can affect adipose tissues, the heart, and the hypothalamus in an endocrine manner^5, 6^. Therefore, we tested the possibility that endocrine Fgf21 from the liver regulates the development of thymocytes. To examine this possibility, we adopted a protein-free diet (PF), which was separately reported to induce upregulation of serum Fgf21 and alter cellularity of thymocytes^24, 25^. Three-week-old WT and *Fgf21* KO male mice were fed with PF for 1 week. As expected, PF feeding intensely induced serum Fgf21 in WT mice (Fig. 8A), and RT-PCR analysis revealed increased expression of *Fgf21* mRNA in the liver, thymus, and white adipose tissue, but not in the skeletal muscle (Fig. 8B). Thymic weight and cellularity of WT and KO mice were significantly decreased by PF-feeding along with body weight and blood glucose level (Fig. 8C–F). We examined the thymocytes using CD4/CD8 and TCRβ/CD69 staining and found that the percentage of the CD4SP, CD8SP, and T3-5 thymocytes were similarly increased by PF feeding in both genotypes (Fig. 8G and H). Similar results were obtained in analysis of PF-feeding with 8-week-old WT and *Fgf21* KO mice (Fig. S5). Interestingly, decreased glycaemic difference between normal chow (NC) and PF feeding in KO mice was smaller than that of WT mice only at 8-week-old (Fig. S5D). These data suggest that Fgf21 induced by protein-malnutrition might not affect the alteration of thymocytes.

## Discussion

Since Kharitonenkov *et al*. reported a biological function of FGF21 in improving lipid and carbohydrate homeostasis, and its potential therapeutic application for diabetes^2^, the interest in FGF21 has focused on its functional analysis within metabolic tissues such as white and brown adipose tissue, as well as in that of the liver, pancreas, and hypothalamus^6^. In addition, Youm *et al*. recently expanded the metabolic benefits of Fgf21 into immunity. They demonstrated that Fgf21 protects against age-related thymic involution and reduces the accumulation of white adipocytes within the thymic space in 12-month-old *Fgf21* transgenic and KO mice^13^. In contrast, our research *in vivo* and *in vitro* revealed the function of Fgf21 in the neonatal and juvenile thymus. Although the difference between WT and *Fgf21* KO mice in the percentage of DN, DP, and SP cells was small and/or not significant, these differences or tendency were reproducible, and FTOC experiments, which seem to reflect development of neonatal stage, also resulted in the same direction (Fig. 6). Neonatal and juvenile *Fgf21* KO mice had impaired thymocyte development without accumulation of perithymic adipocytes (data not shown). Furthermore, the results from FTOC systems support that thymic function of Fgf21 during the juvenile period is independent from the metabolic function of Fgf21 in extrathymic tissues. The decreasing expressions of *Fgfrs* along with thymic development might explain the neonatal- and juvenile-specific phenotype of *Fgf21* KO mice.

Among the FGF family, prior studies demonstrated that FGF7/KGF and Fgf10 can also affect thymic development via Fgfr2b^26–28^. Unlike FGF7 and Fgf10, Fgf21 might regulate thymocyte development without affecting the proliferation of TECs at least in the developmental and juvenile stages (Figs 4 and 6), even though aged *Fgf21* KO mice exhibited accelerated reduction of TECs associated with lipotoxicity of metabolic abnormalities^13^. These data suggest that Fgf21 functions in a manner different from FGF7 and Fgf10 in the thymus. In fact, Fgf members have different specificities and affinities to Fgfr1-4^29^. Fgf7 and Fgf10 preferentially activate Fgfr2b with the support of heparin/heparan sulfate (HS), whereas, Fgf21 binds to and activates Fgfr1c and Fgfr3c, but not Fgfr2b.

Fgf21 is thought to require β-Klotho as a co-receptor for binding to Fgfrs from *in vitro* studies^18, 29, 30^. However, it remains unclear whether β-Klotho is essential for all of Fgf21 bioactivity *in vivo*, because Tomiyama *et al*. demonstrated that Fgf21 signals were transduced in the absence of β-Klotho in adipose tissues^17^. In our experiments, *Klb* ^−/−^ mice did not show impairment of thymocyte development as *Fgf21* KO mice did, although, consistent to earlier reports^13, 27^, expression of *Klb* as well as of *Fgfr* could be detected in TECs and CD45^−^EpCAM^−^ fractions including fibroblasts and endothelial cells (Figs 2, 3 and 5). These data support the existence of Klotho-independent Fgf21 signalling pathway(s) in the thymus^17^. Further studies are required to understand how Fgf21 exerts its physiological activities *in vivo*. On the other hand, 1-week-old *Klb* ^−/−^ mice exhibited reduced ratios of CD4SP and CD8SP cells in splenocytes, but not in thymocytes (Fig. 5H). Since β-Klotho is also essential for the physiological roles of Fgf19^6, 7^, Fgf19 and/or Fgf21 might regulate the migration or proliferation of T cells in the spleen independently of thymopoiesis. This possibility might partially account for the reduction of CD4SP and CD8SP cells in the spleen of Fgf21 KO mice (Fig. 2F).

In addition to the affinity to Klotho, Fgf19/21/23 have characteristic properties distinguishable from canonical Fgfs. They primarily function as endocrine factors and are referred as endocrine Fgfs^29^. Fgf21 is produced in the liver and affects adipose tissues, as well as the heart and hypothalamus, to regulate metabolic homeostasis. Our results for the FTOC experiments indicate that *Fgf21* KO thymus exhibited a reduced number of mature thymocytes in comparison to that of the WT, emphasizing the importance of paracrine or autocrine intrathymic Fgf21 (Fig. 6). Moreover, we examined whether endocrine Fgf21 could regulate thymopoiesis under physiological conditions. To induce endocrine Fgf21 from the liver, we preliminarily tested several metabolic stresses such as fasting, drinking sucrose water, high-fat diet, and PF^24, 31–34^. Among these stresses, PF could induce the highest level of endocrine Fgf21, and only PF could alter the percentage of CD4SP and CD8SP cells accompanied by decreased cellularity as shown in FTOC with FGF21 (Figs 6, 8 and S5). Other stresses could not alter subpopulations of thymocytes despite of relatively high serum Fgf21 (data not shown). Nevertheless, WT and *Fgf21* KO mice exhibited equivalently alter thymocyte subpopulations by PF feeding (Fig. 8G and H). Consequently, we concluded that the alteration of thymocytes under protein malnutrition is independent from Fgf21. Furthermore, considering that overexpression of Fgf21 from the liver under the control of the ApoE promoter, which is estimated to induce exogenous target genes in the liver from the foetal period and extending throughout a lifetime^35, 36^, rescued age-related thymic degradation, but did not affect juvenile thymopoiesis^13^, these data support that endocrine Fgf21 cannot affect development of thymocytes physiologically in the juvenile stage.

The result that rhFGF21 increases apoptosis of immature thymocytes at the selection stage (T3) suggests that Fgf21 may facilitate the selection or apoptosis of developing T cells. On the other hand, we could not detect obvious difference in the thymocyte apoptosis between WT and *Fgf21* KO mice in *vivo* (Fig. S4). This different result between FTOC and in *vivo* thymus might be due to the complexity of apoptotic thymocyte clearance in *vivo* ^37^. Thymic stromal cells, which expressed *Fgfr1-4* and *Klb* predominantly in the thymus, play essential roles in positive and negative selection^23^, suggesting indirect effects of Fgf21 on thymopoiesis via thymic stromal cells. In our study, we could not detect major alteration in thymic structures or gene expressions involved in the functions of thymic stromal cells (Figs 4 and 6). Also, we observed comparable expressions of MHC class II on TECs between WT and *Fgf21* KO mice by flow cytometry (Fig. 4E). There is a slight possibility that Fgf21 directly binds to Fgfrs weakly expressed on the immature thymocytes to control selection of thymocytes. Additional experiments will have to be performed to clearly elucidate the mechanisms how Fgf21 regulates the development of thymocyte directly or indirectly.

In summary, we provide evidence that Fgf21 plays a novel role as an intrathymic cytokine during the neonatal and juvenile stages. Although the functional target cells of thymic Fgf21 remain unclear because of its β-Klotho-independency, our results suggest that Fgf21 secreted from mature mTECs might regulate thymocyte development in a β-Klotho-independent manner.

## Methods

### Mice

All mice were maintained in a light-controlled room (lights on from 0700 to 1900 h). Mice were allowed free access to water and normal chow (MF; 3.6 kcal/g, 12% kcal fat, source: soybean; Oriental Yeast) in normal breeding. The experiments were performed using male mice. *Fgf21* KO (*Fgf21* ^−*/*−^) mice and *Klb* ^−/−^ mice were generated as described^38, 39^, and maintained on a C57BL/6 background. The protein-free diet (PF) feeding experiments were performed with 3- and 8-week-old WT and *Fgf21* KO mice. These mice were fed with normal chow diet (AIN-93G) or PF, in which casein in AIN-93G was replaced with maltodextrin for 1 week. All animal studies were conducted in accordance with International Guiding Principles for Biomedical Research Involving Animals, and approved by the Animal Research Committee of Kobe Pharmaceutical University.

### Flow cytometry and sorting

Thymocytes and splenocytes were prepared by applying pressure with the head of a syringe to the thymus and spleen, and collected through a 70 μm cell strainer. Single-cell suspensions of TECs were prepared by digestion with DNase I and Liberase (Roche) as described previously^9^. These cells were incubated with fluorochrome-conjugated antibodies and lectin, and immunofluorescence was analysed and sorted with a FACSAriaIII (BD Biosciences). Antibodies used were anti-CD45 (A95-1), anti-EpCAM (G8.8), anti-MHC II I-A/I-E (M5/114.15.2), anti-CD4 (RM4-4), anti-CD8a (53–6.7), anti-TCRβ (H57-597), anti-CD69 (H1.2F3), anti-CD62L (MEL-14), anti-CD24 (M1/69), anti-Ly51 (BP-1), anti-CD31 (MEC13.3), and anti-CD140a (APA5). Fluorescein isothiocyanate–conjugated Ulex Europaeus Agglutinin (UEA-I) lectin was used as a marker for mTECs. Apoptosis was detected by Annexin-V staining, according to the manufacturer’s protocol (BD Biosciences).

### Quantitative RT-PCR

RNA was purified using RNeasy (Qiagen) before first-strand cDNA synthesis using the ReverTra Ace qPCR RT kit (Toyobo). Quantitative-PCR using Thunderbird SYBR qPCR mix (Toyobo) was performed. 18S ribosomal RNA levels were used as an internal control.

### Immunohistochemistry

Fresh frozen thymic sections (12 μm) from 4-week-old mice were fixed with 4% PFA/PBS, and blocked with 1% BSA/PBS. The sections were then incubated with primary antibodies for 16 hour at 4 °C followed by incubation with fluorescence-conjugated secondary antibodies. Images of stained tissues were acquired with an LSM700 inverted confocal microscope (Carl Zeiss). To determine the ratio of TEC regions, four sections from each of 8 thymi per genotype were measured using imageJ software.

### Blood Parameter Analysis

Blood samples were obtained from the mice. Serum Fgf21 levels were measured using a mouse FGF-21 Quantikine ELISA Kit (R&D systems). Blood glucose levels were measured with a Glutest Neo Alpha kit (SANWA KAGAKU).

### Fetal thymus organ cultures (FTOCs)

Foetal thymic lobes were dissected from mouse embryos at E15.5 gestational stage and placed on Millicell 0.4-μm plate inserts (Millipore). Millicell discs were overlaid on serum-free AIMV medium (Life Technologies). Medium containing 500 ng/ml of recombinant human FGF21 (R&D Systems) was replaced at 3-day intervals. After 14 days, thymic lobes were dissociated and stained for flow cytometric analysis. For the 2-deoxyguanosine-treated FTOC (2DG-FTOC) experiment, thymic lobes (E15.5) were cultured in dGuo (1.35 mM) for 4 days, and after washing, they were re-cultured with FGF21 for an additional 4 days.

### Statistical analysis

Data were analysed using Prism software (GraphPad Software). The presented data are expressed as means ± SD. One-way ANOVA with the Tukey’s post hoc test was used for analysis of multiple groups, and a Student’s t test was used to compare the 2 groups. Flow cytometry data were analysed using a 2-way ANOVA. P < 0.05 was considered statistically significant.

## Electronic supplementary material

Supplementary information
