# Supplementary material for: Fgf21 regulates T-cell development in the neonatal and juvenile thymus
Source: Sci Rep. 2017 Mar 23;7:330. doi: 10.1038/s41598-017-00349-8 (PMC5428243; doi:10.1038/s41598-017-00349-8)
Supplement: Supplementary file 1 — Supplementary information [file 41598_2017_349_MOESM1_ESM.pdf]

Fgf21 regulates T-cell development in the neonatal and juvenile thymus

Yoshiaki Nakayama[1]†, Yuki Masuda[1]†, Hiroya Ohta[1,2], Tomohiro Tanaka[3,4], Miwa Washida[4], Yo-ichi Nabeshima[4], Ayumi Miyake[5], Nobuyuki Itoh[3,5], Morichika Konishi[1,5]*

[1]Department of Microbial Chemistry, Kobe Pharmaceutical University, Kobe, Japan; [2]Department of Molecular Medicine and Metabolism, Research Institute of Environmental Medicine, Nagoya University, Nagoya, Japan; [3]Medical Innovation Center, Graduate School of Medicine, Kyoto University, Kyoto, Japan; [4]Laboratory of Molecular Life Science, Foundation for Biomedical Research and Innovation, Kobe, Hyogo, Japan; [5]Department of Genetic Biochemistry, Kyoto University Graduate School of Pharmaceutical Sciences, Sakyo, Kyoto, Japan.

*Correspondence and requests for materials should be addressed to M. K. (email: mkonishi@kobepharma-u.ac.jp)

†These authors contributed equally to this work.

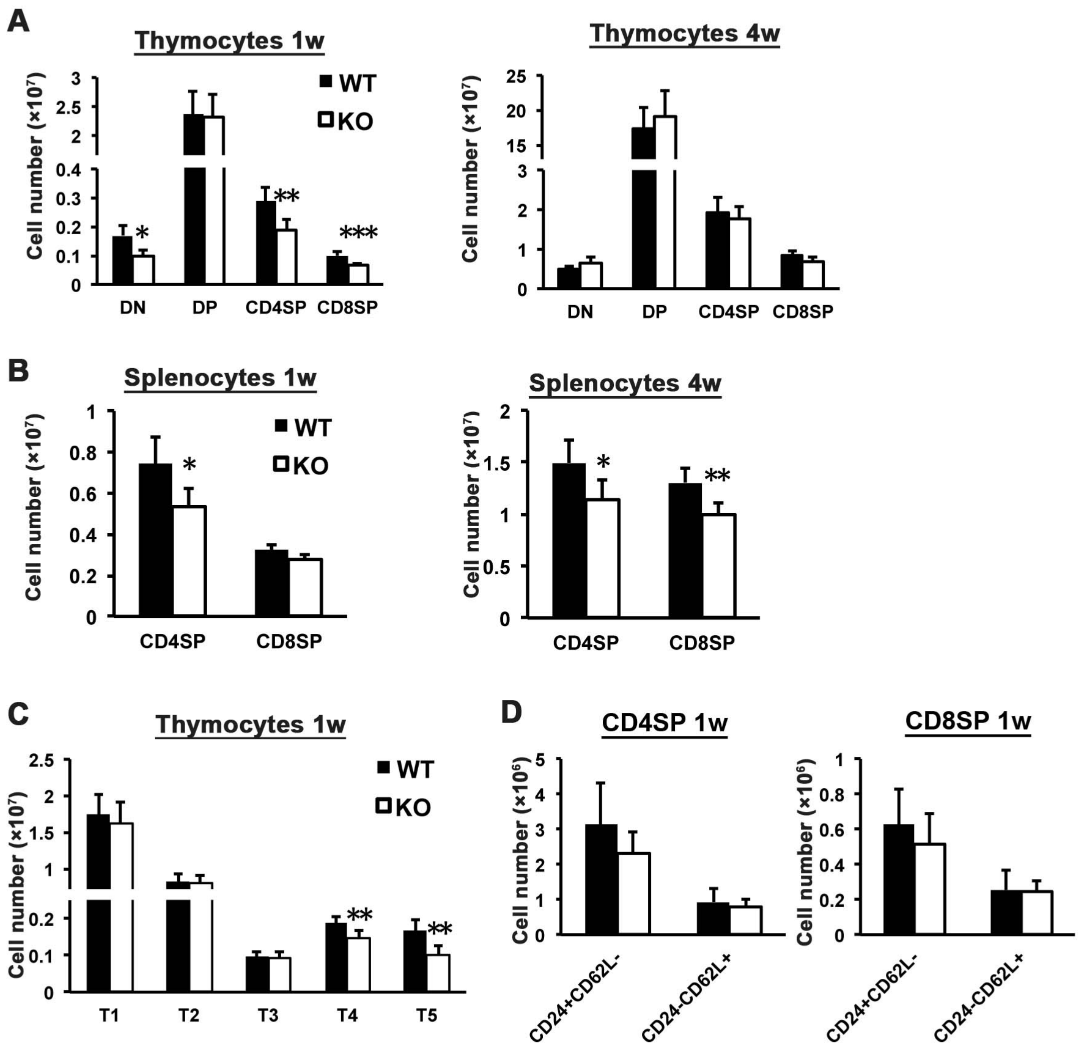

Supplementary Figure S1. Mature T cell populations are decreased in *Fgf21* KO mice. (A and B) Thymocytes and splenocytes from WT and *Fgf21* KO mice were stained with anti-CD4 and anti-CD8 mAb. Charts show the cellularity of DN, DP, CD8SP, and CD4SP cells from 1- and 4-week-old WT and *Fgf21* KO mice. (C) Thymocytes from 1-week-old WT and *Fgf21* KO mice were defined by TCRβ and CD69 levels and subdivided into 5 subsets (T1; $TCR\beta^{-}CD69^{-}$, T2; $TCR\beta^{int}CD69^{-}$, T3; $TCR\beta^{int}CD69^{+}$, T4; $TCR\beta^{hi}CD69^{+}$, and T5; $TCR\beta^{hi}CD69^{-}$). Charts show the cellularity of the 5 subsets from 1-week-old WT and *Fgf21* KO mice. (D) Gated CD4SP and CD8SP cells from 1-week-old WT and *Fgf21* KO mice were defined by CD62L and CD24 levels and subdivided into $CD24^{+}CD62L^{-}$ immature and $CD24^{-}CD62L^{+}$ mature SP cells. Charts show the cellularity of $CD24^{+}CD62L^{-}$ immature and $CD24^{-}CD62L^{+}$ mature SP subsets from 1-week-old WT and *Fgf21* KO mice. All data shown are the mean ± SD from ≧ 6 mice per genotype from 2 independent experiments. *: $P < 0.05$, **: $P < 0.01$ versus WT mice.

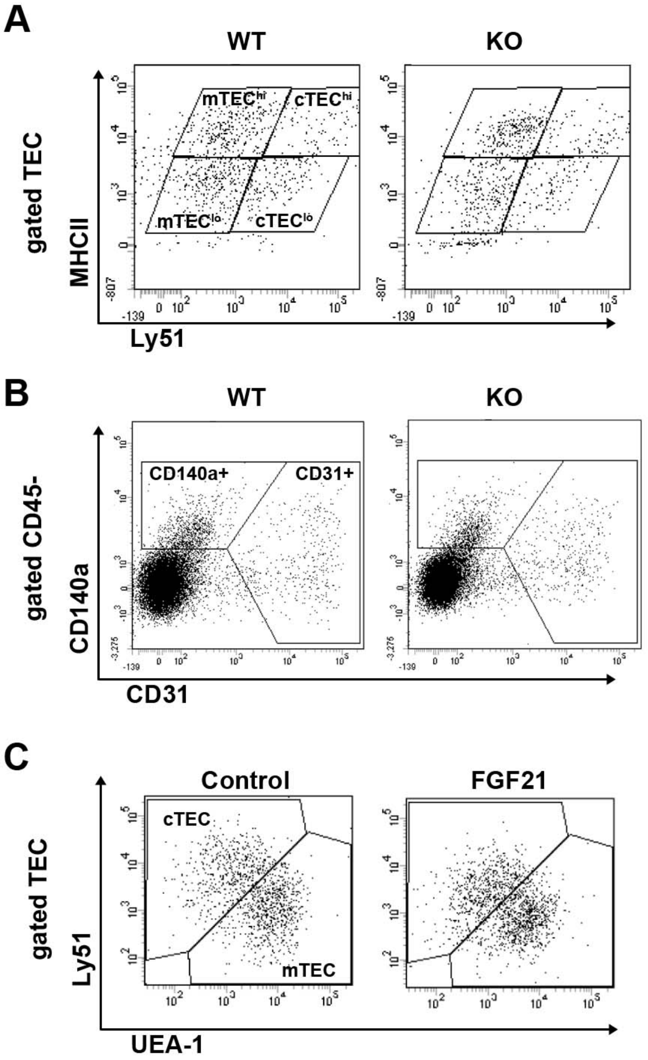

Supplementary Figure S2. Thymic stromal cells were subdivided by flow cytometry. (A and B) Enzymatically digested thymic cell suspensions from 1-week-old WT and *Fgf21* KO mice were assessed by flow cytometric analysis. Panels show representative histograms. (A) Gated $CD45^{-}EpCAM^{+}$ TEC were subdivided into mTEChi ($Ly51^{-}MHC^{high}$), mTEClo ($Ly51^{-}MHC^{low}$), cTEChi ($Ly51^{+}MHC^{high}$), and cTEClo ($Ly51^{+}MHC^{low}$) subpopulations. (B) $CD45^{-}CD140a^{+}$ fibroblasts and $CD45^{-}CD31a^{+}$ endothelial cells were discriminated from gated $CD45^{-}$ thymic stromal cells. (C) Enzymatically digested thymic cell suspensions from FTOC thimi with or without recombinant human FGF21 protein (500 ng/ml) for 14 days. Gated $CD45^{-}EpCAM^{+}$ TEC were divided into mTEC ($UEA\text{-}1^{+}$) and cTEC ($Ly51^{+}$) subpopulations.

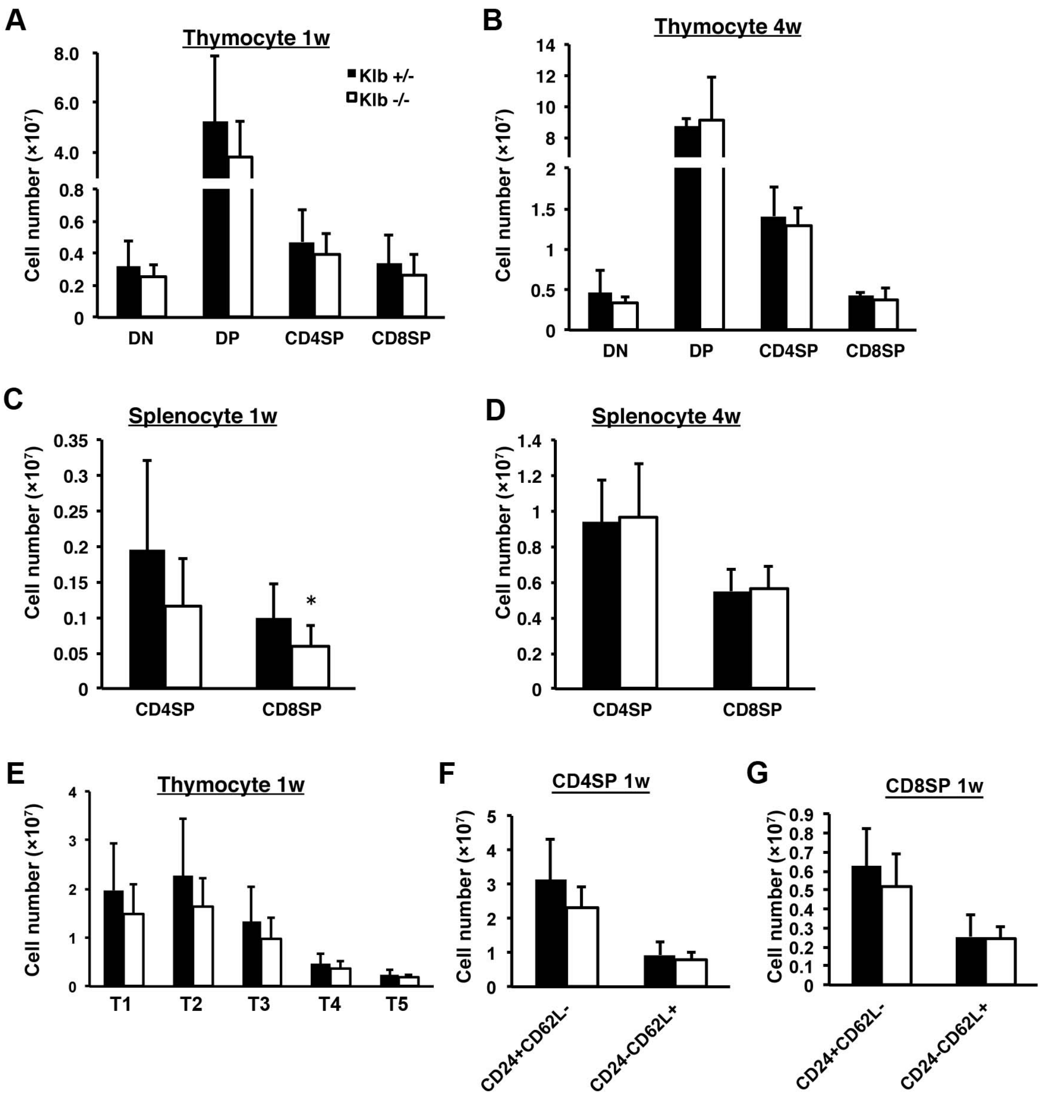

Supplementary Figure S3. ***Klb*** **is not involved in the maturation of thymocytes.** (A–D) Thymocytes and splenocytes from 1- and 4-week-old $Klb^{+/-}$ and $Klb^{-/-}$ mice were stained with anti-CD4 and anti-CD8 mAb. Charts show the cellularity of DN, DP, CD8SP, and CD4SP from 1- and 4-week-old $Klb^{+/-}$ and $Klb^{-/-}$ mice. (E) Charts show the cell number of the 5 subsets defined by TCRβ/CD69 staining from 1-week-old $Klb^{+/-}$ and $Klb^{-/-}$ mice. (E and F) Charts show the cellularity of $CD24^{+}CD62L^{-}$ immature and $CD24^{-}CD62L^{+}$ mature SP subsets from gated CD4SP (F) and CD8SP cells (G) of 1-week-old $Klb^{+/-}$ and $Klb^{-/-}$ mice. All data shown are the mean ± SD from ≧ 8 mice per genotype. *: $P<0.05$, **: $P<0.01$ versus $Klb^{+/-}$ mice.

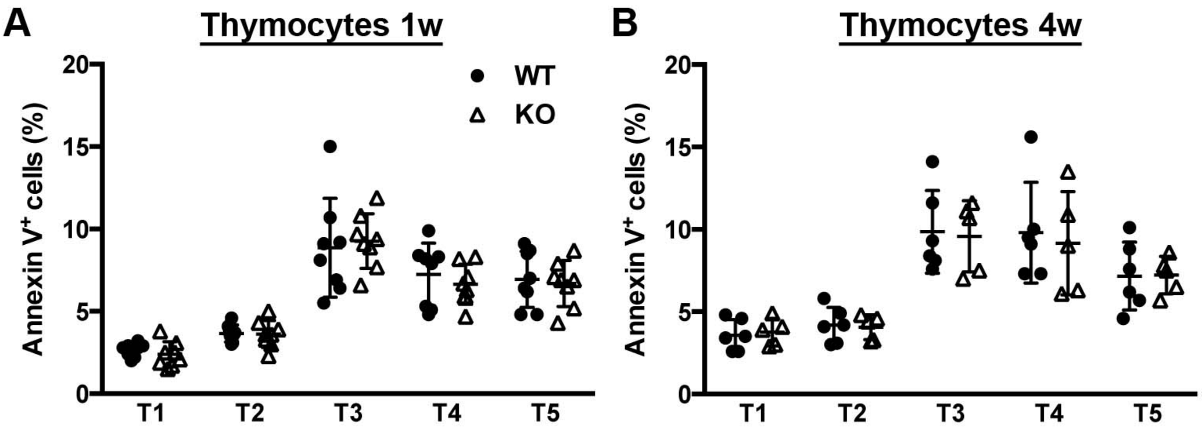

Supplementary Figure S4. *Fgf21* KO mice did not show obvious change in apoptosis of immature thymocytes. Thymocytes from 1-week-old (A) and 4-weeks-old (B) WT and *Fgf21* KO mice were stained with annexin V to detect apoptotic T cells by flow cytometry. Graph showing the analysis of the percentage of annexin V^+ cells in thymocyte. All data shown are the mean ± SD from ≧ 5 mice per genotype.

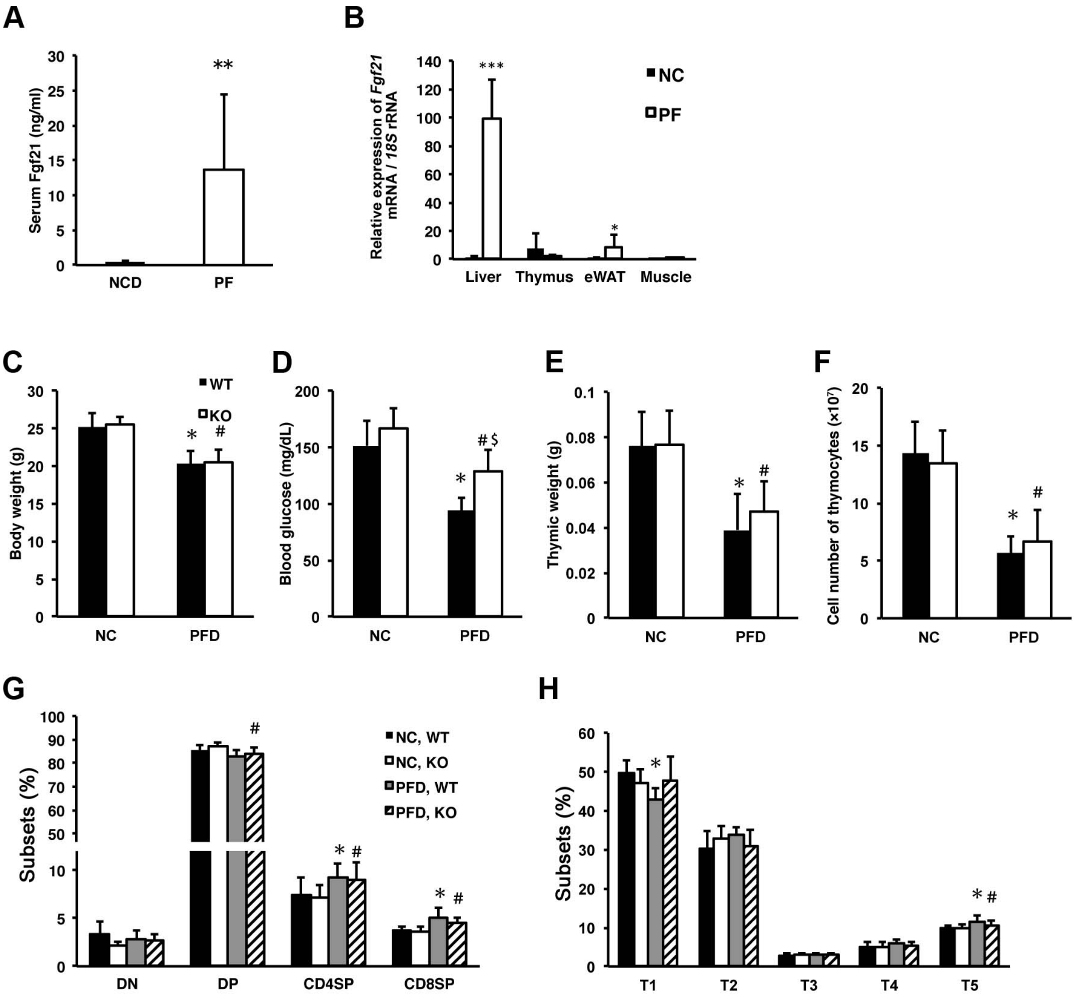

Supplementary Figure S5. Endocrine Fgf21 from the liver is dispensable for thymic change by protein malnutrition. (A) Serum Fgf21 levels of C57BL/6 mice fed with normal chow diet (NC) or protein-free diet (PF) were determined with ELISA assays. Data shown are the mean ± SD from ≧ 10 mice. **: $P < 0.01$ versus NC. (B) Relative expression levels of *Fgf21* mRNA in the liver, thymus, epididymal white adipose tissue (eWAT), and skeletal muscle from mice fed with NC or PF. Data shown are the mean ± SD from ≧ 5 mice.* : $P < 0.05$, ***: $P < 0.001$ versus NC. (C-F) Body weight, blood glucose, thymic weight, and thymic cell number of WT and *Fgf21* KO mice fed with NC or PF. Data shown are the mean ± SD from ≧ 6 mice.* : $P < 0.05$,$^{\#}$: $P < 0.05$ versus WT and *Fgf21* KO mice fed with NC, respectively. $^{\$}$: $P < 0.05$ versus WT mice fed with PF. (G and H) Thymocytes from WT and *Fgf21* KO mice fed with NC or PF were stained with anti-CD4 and anti-CD8 mAb (G), or anti-TCRβ and anti-CD69 mAb (H). All data shown are the mean ± SD from ≧ 6 mice. * : $P < 0.05$,$^{\#}$: $P < 0.05$ versus WT and *Fgf21* KO mice fed with NC, respectively.
